# Generation of a Live-Attenuated Strain of Chikungunya Virus from an Indian Isolate for Vaccine Development

**DOI:** 10.3390/vaccines10111939

**Published:** 2022-11-16

**Authors:** Sreeja R. Nair, Rachy Abraham, Easwaran Sreekumar

**Affiliations:** 1 Molecular Virology Laboratory, Rajiv Gandhi Centre for Biotechnology (RGCB), Thiruvananthapuram 695 014, Kerala, India; 2 Bioscience Research and Training Centre (BRTC), Kerala Veterinary and Animal Sciences University, Bio360 Life Sciences Park, Thonnakkal, Thiruvananthapuram 695 317, Kerala, India; 3 Institute of Advanced Virology (IAV), Bio360 Life Sciences Park, Thonnakkal, Thiruvananthapuram 695 317, Kerala, India

**Keywords:** chikungunya, CHIKV, live-attenuated vaccine, ECSA strain, human astrocytoma cell line, U-87 MG cells

## Abstract

Chikungunya virus (CHIKV) re-emergence in the last decade has resulted in explosive epidemics. Along with the classical symptoms of fever and debilitating arthralgia, there were occurrences of unusual clinical presentations such as neurovirulence and mortality. These generated a renewed global interest to develop prophylactic vaccines. Here, using the classical approach of virus attenuation, we developed an attenuated CHIKV strain (RGCB355/KL08-p75) for the purpose. Repeated passaging (75 times) of a local clinical isolate of ECSA lineage virus in U-87 MG human astrocytoma cells, an interferon-response-deficient cell line, resulted in efficient adaptation and attenuation. While experimental infection of 3-day old CHIKV-susceptible BALB/c pups with the parent strain RGCB355/KL08-p4 resulted in death of all the animals, there was 100% survival in mice infected with the attenuated p75. In adult, immunocompetent, CHIKV-non-susceptible C57BL/6 mice, inoculation with p75 induced high antibody response without any signs of disease. Both p4 and p75 strains are uniformly lethal to interferon-response-deficient AG129 mice. Passive protection studies in AG129 mice using immune serum against p75 resulted in complete survival. Whole-genome sequencing identified novel mutations that might be responsible for virus attenuation. Our results establish the usefulness of RGCB355/KL08-p75 as a strain for vaccine development against chikungunya.

## 1. Introduction

Chikungunya virus (CHIKV; family *Togaviridae*, genus Alphavirus) is an arthropod-borne virus transmitted to humans mainly by the *Aedes* species [1,2]. The disease presents as an acute illness in infected patients, characterized by a high fever, severe and debilitating joint pain often associated with a rash, muscle pain, headache, nausea and fatigue [3]. Extensive chikungunya outbreaks were described earlier in Southeast Asia and especially in India during the 1950s and 1960s [4,5]. The re-emergence of the disease in 2004, the first after 32 years of quiescence, documented with massive disease outbreaks in the islands of the Indian Ocean and Indian subcontinent, has subsequently spread to many parts of the world [6,7]. These newer outbreaks were associated with numerous other complications including cardiomyopathies, neurological disease, multi-organ failure and death [8,9,10]. Currently, CHIKV has been identified in over 114 countries and territories throughout the globe, affecting millions of people [11].

Even though the CHIKV resurgence has a wide geographic spread and extreme economic impact, there are currently no licensed vaccines or approved therapeutics against the disease. Attempts to develop a CHIKV vaccine started as early as the 1960s, with formalin-inactivated virus preparations [12]. Strategies for the development of live-attenuated vaccines were given preference because of the decreased production costs and the reduction in the possible risks associated with handling large quantities of un-attenuated viruses prior to inactivation. The first CHIKV-attenuated strain, TSI-GSD-218 (strain 181/clone25, derived from Asian lineage strain AF15561), which reached Phase II clinical trials, was developed during the 1980s by the US Army Medical Research Institute for Infectious Diseases (USAMRIID). This live-attenuated strain was generated by serial plaque-to-plaque passaging of a wild-type Thailand CHIKV strain in human lung cells, MRC-5 cells, and was proved to be highly immunogenic in humans [13,14]. However, it resulted in mild, transient arthralgia in a subsection of the vaccine recipients, which pointed out its tendency of reversion to virulence [15]. Currently, Phase III trials of a virus-like particle (VLP)-based VRC-CHKVLP059-00-VP (PXVX0317), sponsored by Emergent BioSolutions [16,17,18,19,20]; a live-attenuated Δ5nsP3 VLA1553-301, of Butantan Institute and Valneva Austria GmbH [21,22,23,24]; a Phase II/III adaptive seamless designed trial of inactivated virus BBV87, of Bharat Biotech International Limited [25]; and a Phase II trial of measles virus vector-based MV-CHIK, of Themis Bioscience GmBH [26,27,28,29,30], are ongoing. Nearly 30 novel CHIKV vaccine candidates are presently under development in various strategies based on inactivated, live-attenuated, viral-vectored, chimeric, virus-like particles, subunit proteins, DNA and mRNA [31,32,33,34]. A comprehensive list of vaccine candidates that were considered for clinical and preclinical trials until 2022 are listed out in Table 1.

Genome sequencing of CHIKV isolates has identified three geographically associated genotypes: Asian; East, Central and South African (ECSA); and West African. However, there is only one known serotype [7,77]. Additionally, two important sub-lineages emerged during the epidemics of the past two decades: the Indian Ocean Lineage (IOL) from the ECSA genotype during the Indian Ocean outbreak of 2005 [78,79], and the Asian/American lineage within the Asian genotype, originated during 2013 Caribbean outbreak [80,81]. Studies have indicated that the protective immune response against CHIKV is not genotype-specific, leading to cross-lineage protective immunity against all others [82,83]. However, a recent study has shown that maximum neutralization by anti-CHIKV antibodies is exhibited against viral strains of the same lineage [84]. This has pointed to the need for using homotypic strains for making more efficient anti-CHIKV vaccines suitable for different geographical areas depending on the CHIKV lineage prevalent in the region.

The ECSA genotype is the most circulating strain in the Indian subcontinent and many parts of South Asia, and hence most of the recent vaccine candidates are based on the ECSA strain (Table 1). In the present study, we sought to develop an attenuated strain of the CHIKV ECSA genotype for supporting the development of live-attenuated vaccines. 

## 2. Materials and Methods

### 2.1. Cell Culture and Viruses

Vero cells (African green monkey kidney cells; National Centre for Cell Sciences, Pune, India) and U-87 MG cells (ATCC- HTB14; American Type Culture collection) were grown in DMEM (Dulbecco’s modified Eagle’s medium; Gibco, Thermo Fisher Scientific, Waltham, MA, USA) supplemented with 10% heat-inactivated fetal bovine serum (FBS) and 1× antibiotic-antimycotic mixture (all from Sigma-Aldrich, Bangalore, India). Cultures were incubated at 37 °C in a humidified atmosphere containing 5% CO_2_. 

The virus strain used, a 2008 human isolate from Kerala, India, designated as RGCB 355/KL08, was described earlier [85]. It was passaged four times in Vero cells and was designated as RGCB 355/KL08-p4 (p4). RGCB 355/KL08-p4 was further used to infect U-87MG cells and was passaged 75 times to produce the attenuated strain designated as RGCB 355/KL08-p75 (p75). The replication kinetics of the virus stocks (p4 and p75) in U-87 MG cells were assessed on Vero cell monolayers by plaque assay as previously described [85].

### 2.2. Immunofluorescence Staining

Cells were grown on glass cover slips and infected at MOI 1. At different times post-infection, cells were fixed using 4% paraformaldehyde in phosphate-buffered saline (PBS; pH 7.2; Gibco) for 15 min at 4 °C and washed three times with PBS. The cells were permeabilized with 0.2% TritonX-100 for 10 min at room temperature, washed with PBS and blocked by incubating in PBS containing 8% normal goat serum for 1 h at 37 °C. After washing with PBS, cells were incubated in a 1:50 dilution of primary antibody (in-house rabbit anti-CHIKV polyclonal serum against recombinant E2 protein) for 2 h. After three washes with PBS, cells were incubated with a 1:2000 dilution of Alexafluor 488-conjugated anti-rabbit IgG (Invitrogen). Nucleus morphology was revealed by DAPI staining (final concentration: 1 μg/mL). Cover slips were mounted on glass slides and images were captured using a confocal microscope (Nikon A1R; Nikon Instruments, Melville, NY, USA), with identical settings for the infected cells and the controls, and images were analyzed with NIS elements software.

### 2.3. Animal Experiments

#### 2.3.1. Ethics Statement

All animal experiments were carried out strictly following the approved study protocol of Institutional Animal Ethics Committee (IAEC) of Rajiv Gandhi Centre for Biotechnology (RGCB) (IAEC/502/ES/2015). Inbred un-weaned 3-day-old BALB/c mice of either sex; 4- to 6-week-old C57BL/6 of either sex; and 3- to 4-week-old AG129 (Interferon-α/β and -γ receptor knockout IFNα/β/γR-/-, B&K Universal, Grimston, UK) mice of either sex, bred and housed under specific pathogen-free conditions at the Animal Research Facility of RGCB, were used in the study. All animals were provided with pellet diet and water ad libitum.

#### 2.3.2. CHIKV Virus Infection in Mouse Models

Timed pregnant BALB/c mice were maintained in the animal facility and 3-day-old BALB/c pups (n = 24 from 3 litters for each group) as well as 3- to 4-week-old AG129 mice (n = 6 for each group) were infected subcutaneously in the loose skin on the back with 10^2^ Plaque Forming Unit (PFU) and 10 PFU of the CHIKV p4 and p75 in 50 µL of DMEM. For mock infection, 50 µL of DMEM without virus was injected. Following infection, mice were monitored daily at a regular interval of 24 h until day 10 post infection (dpi) for morbidity/mortality, and clinical signs were scored daily from 0 to 10 dpi. For BALB/c pups, clinical signs were scored as 0—normal behavior, 1—generalized cachexia, 2—walking difficulty and patchy alopecia, 3—epileptic seizures and ataxic gait, and 4—dead, while for AG129 mice the clinical signs were scored as 0—normal behavior, 1—lethargy and starting of weight loss, 2—hunched back posture, 3—convulsions, and 4—dead. 

#### 2.3.3. Mice Immunization

Two groups (n = 3 each) of 4- to 6-week-old C57BL/6 mice were injected subcutaneously with 10 PFU of the CHIKV p75 in DMEM with 2% FBS. Booster injections with same formulation were given on day 28 and day 56. Serum samples were collected from individual mice at weekly intervals until day 84 post-infection. 

#### 2.3.4. Enzyme Linked Immunosorbent Assay

The predicted antigenic region of the CHIKV E2 protein was identified (aa 1–62, ∆E2) based on the hydrophilicity profile, and it was cloned and expressed as a recombinant protein in BL-21 DE3 *E. coli* using a pET-32 (Novagen) expression system. For indirect ELISA, a 96-well microtiter plate (Nunc-Immuno Plate, MaxiSorp; Thermo Fisher Scientific, Waltham, MA, USA) was coated with recombinant ∆E2 protein at 5 µg/µL in coating buffer (50 mM carbonate/bicarbonate buffer, pH 9.6), and kept overnight at 4 °C. Plates were blocked with 2% BSA (Sigma, St. Louis, MO, USA) in PBS at room temperature for 1 h. The wells were incubated with a 1:100 dilution of the serum in triplicate wells (100 µL/well) at room temperature for 1 h. Sufficient washes with PBS-Tween were performed after each step. The bound antibodies were detected by HRP-conjugated goat anti-mouse IgG (1:30,000, Sigma) and developed using TMB substrate (Sigma). The reaction was stopped using 2N HCl and plates were read on a microplate reader at 415 nm. 

#### 2.3.5. Plaque Reduction Neutralization Tests 

Serum samples were heat inactivated at 56 °C for 30 min and then serially diluted 2-fold in DMEM without FBS. Diluted test sera were incubated with an equal volume of p75 variant at a concentration of 100 PFU/mL, at 37 °C and 5% CO_2_ for 1.5 h. Confluent Vero cell monolayer in 24-well plates was incubated with 100 μL of virus–sera mixture at 37 °C with 5% CO_2_ for 1 h, after which the inoculum was removed and the cells were overlaid with 1 mL of 1.5% carboxymethyl cellulose (Sigma) in 2× DMEM with 2% heat-inactivated FBS. The plates were incubated at 37 °C with 5% CO_2_ for 48 h, fixed with 30% formalin (Himedia, Thane, India) in PBS followed by staining with 0.05% crystal violet solution (Sigma). The number of plaques was counted and the PRNT_90_ titer was calculated and defined as the dilution of serum required to neutralize 90% or more of the virus infection.

#### 2.3.6. Passive Protection Assays

Two groups of 3- to 4-week-old AG129 (n = 3 each) mice were infected subcutaneously with 10 PFU of p75 variant, followed by immediate intraperitoneal administration of 200 µL pooled anti-CHIK immune serum collected from the mice previously immunized with 10 PFU of the attenuated strain p75. Control mice received normal mouse serum. Animals were observed daily for 10 days for scoring morbidity and mortality. 

### 2.4. Whole-Genome Sequencing 

Whole-genome characterization of p4 and p75 was conducted as previously mentioned [86]. The region spanning the whole genome was amplified in small fragments by reverse transcription (RT)-PCR, which was performed under the conditions 42 °C for 30 min and 35 cycles of thermal cycling, which included denaturation at 95 °C for 1 min, annealing at 55 °C for 1 min, and an extension at 68 °C for 3 min. The amplified products were purified using Illustra GFX PCR purification kit (GE Healthcare, Buckinghamshire, UK) and subjected to bi-directional sequencing with overlapping primers using the Big-dye Terminator Cycle sequencing kit in an ABI 3730 Genetic Analyzer automated DNA sequencer (PE Applied Biosystems, Foster City, CA, USA). The sequence contigs were assembled using CAP contig assembly program in BioEdit software.

### 2.5. Statistics

Data from 3 independent experiments of at least 3 mice per group were used. Survival was compared using Kaplan–Meyer survival curves (log rank test). Association measures of survival, such as the hazard ratio and its 95% CI, were calculated using the log rank test. Differences between groups during the course of infection were determined using two-way ANOVA and Bonferroni post-tests. Differences between groups at a single time point were determined using an unpaired, two-tailed Student’s t-test with a 95% confidence interval. Data were analyzed with GraphPad Prism version 7 software (GraphPad software, La Jolla, CA, USA). 

## 3. Results

### 3.1. Infectivity Phenotype of the Wild-Type and Attenuated CHIKV Strains

**Figure 1:**
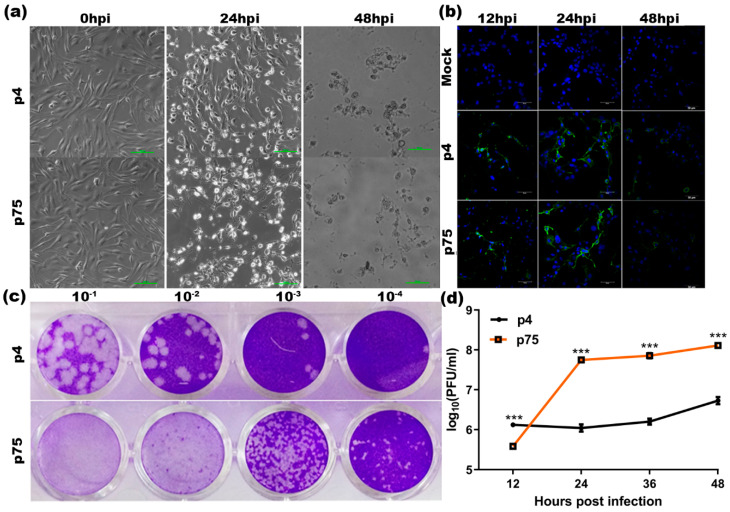
Comparison of infectivity phenotype of the wild-type virus (p4) and the attenuated strain virus (p75) (**a**) Bright-field microscopic images showing the cytopathic changes. U-87 MG cells were infected with either p4 or p75 at MOI 1 and incubated for different time points post-infection. The incidence of cytopathic effect was observed under a microscope. Representative images were acquired at a magnification of 10×; Scale bar—500 μm (**b**) Immunofluorescence detection of p4 and p75 virus infection. Mock-infected and virus-infected U-87 MG cells at MOI 1 were incubated for 24 hpi and 48 hpi. Cells were fixed using 4% paraformaldehyde and subjected to immunofluorescence analysis. Infection was detected using an in-house anti-CHIKV E2 envelope protein rabbit polyclonal serum at 1:50 dilution and using the secondary antibody, Alexafluor 488 anti-rabbit IgG. Representative images were acquired at a magnification of 10×; Scale bar—500 μm (**c**) Plaque morphology of p4 and p75 viruses. A monolayer culture of the Vero cells was grown in 24-well plate and infected with either p4 or p75 in 10-fold dilutions. The cells were overlaid with 3% carboxymethyl cellulose in 2× DMEM with 2% FBS, after removing the un-adsorbed viruses and a PBS wash. The cells were fixed at 48 hpi and stained with 0.02% crystal violet to visualize the plaques. (**d**) Replication kinetics of p4 and p75 virus. U-87 MG cells were infected at MOI 1 and the culture supernatants were collected at different times post-infection and titrated on a confluent Vero cell monolayer by plaque assay. The number of plaques were counted to find the PFU/mL. Average number of plaques from 3 independent experiments performed in duplicate wells with countable plaques (<100) were used to plot the graph. Two-way ANOVA with Bonferroni test to compare the replicate means by row was applied. *** *p* value corresponds to *p* < 0.001 (p4 vs. p75). Data were pooled from 3 independent experiments and are presented as the means ± SEM.

RGCB 355/KL08, a whole-genome characterized human isolate from the 2008 outbreak that belonged to the East/Central/South African genotype [85,86] was passaged four times in Vero cells and was labelled as parent virus RGCB 355/KL08-p4 (p4) for this study. U-87 MG cells, human astrocytoma cells, were infected with RGCB 355/KL08-p4 and sequential passaging was continued until the 75th passage to produce the live-attenuated CHIKV strain, RGCB 355/KL08-p75 (p75). The replication kinetics of the parent virus (p4) and the attenuated strain (p75), as well as the changes in the cell morphology upon infection, were compared by infecting the U-87 MG cells with both of the viral variants at MOI 1. The incidence of cytopathic effect was early for p75 and was evident with changes such as rounding and intracytoplasmic granulation, as seen from the bright field as well as the immunocytochemistry images (Figure 1a,b). Plaque morphology was consistent throughout the experiment, with p75 forming small discrete plaques, as compared to p4, which had larger and more diffused plaques (Figure 1c). To understand the replication efficiency of the viral variants, the supernatants at different times post infection were collected and titrated in Vero cells through plaque assay. The viral titer of p75 started to peak at 24 h post-infection (hpi) with a 2-log increase (p75 vs. p4 *** *p* < 0.001) and then plateaued until 48 hpi (36 hpi and 48 hpi *** *p* < 0.001), whereas p4 replicated gradually and peaked only at 48 hpi.

### 3.2. Evaluation of CHIKV p75 Attenuation in Mouse Models

**Figure 2:**
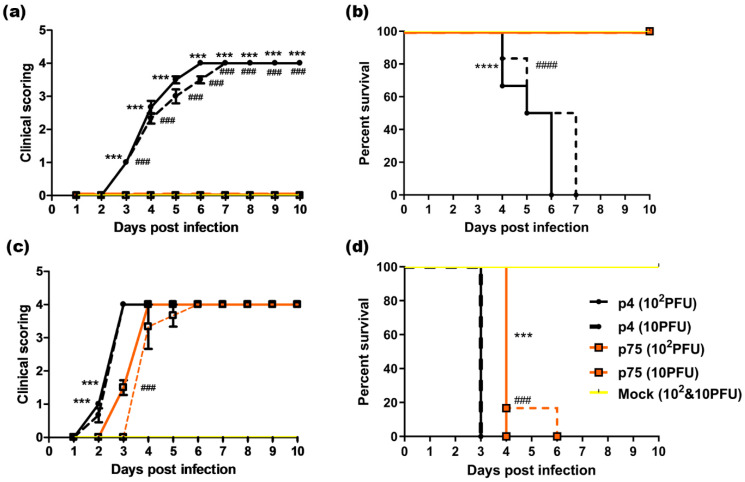
In vivo evaluation of infectivity of p4 and p75 CHIKV strains in susceptible mice. Neonatal, 3-day-old BALB/c mice (n = 24/group) as well as 3- to 4- week-old AG129 mice (n = 6/group) were infected subcutaneously with 10^2^ (solid line) and 10 PFU (dashed line) of p4 and p75. Mock-infected group received the same amount of DMEM (n = 24/gp for BALB/c and 6/group for AG129) and is shown in yellow line with a dash. Data were pooled from 3 independent experiments and are presented as the means ± SEM. (**a**) Clinical scoring of infected BALB/c mice. The appearance of signs of disease in mice was monitored daily. Clinical score scale: 0—normal behavior, 1—generalized cachexia, 2—walking difficulty and patchy alopecia, 3—epileptic seizures and ataxic gait, and 4—dead. Two-way ANOVA with Bonferroni test to compare the replicate means by row was applied. *** *p* value corresponds to *p* < 0.001 (p4 vs. p75 inoculated with 10^2^ PFU) and ### *p* value corresponds to *p* < 0.001 (p4 vs. p75 inoculated with 10 PFU). (**b**) Survival graph of infected BALB/c mice. Survival was compared using Kaplan–Meier survival curves (log rank test). **** *p* < 0.0001 (p4 vs. p75 inoculated with 10^2^ PFU) and #### *p* < 0.0001 (p4 vs. p75 inoculated with 10 PFU). (**c**) Clinical scoring of infected AG129 mice. The appearance of signs of disease in mice was monitored daily. Clinical score scale: 0—normal behavior, 1—lethargy/weight loss, 2—hunched back posture, 3—convulsions, and 4—dead. Two-way ANOVA with Bonferroni test to compare the replicate means by row was applied. *** *p* value corresponds to *p* < 0.001 (p4 vs. p75 inoculated with 10^2^ PFU) and ### *p* value corresponds to *p* < 0.001 (p4 vs. p75 inoculated with 10 PFU). (**d**) Survival graph of infected AG129 mice. Survival was compared using Kaplan–Meier survival curves (log rank test). *** *p* < 0.001 (p4 vs. p75 inoculated with 10^2^ PFU) and ### *p* < 0.001 (p4 vs. p75 inoculated with 10 PFU).

We had previously confirmed symptomatic CHIKV infection in neonatal 3-day-old BALB/c mice [87,88]. Hence, the same model was used for the study. Inoculation of the wild-type virus (p4) in neonatal BALB/c (n = 24) at a dose of 10^2^ PFU resulted in the onset of clinical signs of lethargy and generalized cachexia on day 3 (clinical score—1). The disease progressed with patchy alopecia and walking difficulties (clinical score—2) by day 4, with convulsive movements, epileptic seizures and ataxic gait (clinical score—3) on day 5 and finally death (clinical score—4) in 5–6 days. With a lower dose of 10 PFU (n = 24), the infection started on day 3 itself but progressed more slowly, with death delayed by a day. The mean days of death (MDOD) for mice when inoculated with 10^2^ PFU and 10 PFU of p4 were 5.2 and 5.8 days (Figure 2a,b). In contrast, infection of neonatal BALB/c with the attenuated strain (p75, n = 24) with the two different doses (10^2^ PFU and 10 PFU) progressed without any obvious symptoms and resulted in no morbidity or mortality until the last day of observation (day 10) (Figure 2a,b).

Type I interferons have a distinct protective role against acute CHIKV infection [89], and hence, in immunocompetent adult mice, virus replication is restricted faster, and the animals do not develop apparent infection or lethality. So, in order to evaluate the infectivity of the attenuated strain in adult animals, we used AG129, an IFN-α/β/γ receptor, to knock out mice. Inoculation of AG129 (n = 6/group) mice with 10^2^ and 10 PFU of either wild-type virus or the attenuated strain p75 resulted in infection and death (Figure 2c,d). The disease in both groups progressed rapidly with lethargy and start of weight loss (clinical score—1), hunched back posture (clinical score—2), with convulsive movements (clinical score—3) and finally death (clinical score—4). The mortality was delayed by one day in the mice group inoculated with the attenuated strain as compared to the wild-type virus at both doses of virus inoculation. The MDOD for mice when inoculated with 10^2^ PFU and 10 PFU of p4 was 3 days, while for the ones with p75 it was 4.3 days for 10 PFU and 4 days for 10^2^ PFU. The hazard ratio upon infection with p4 and p75 viruses in AG129 mice for any dose given was calculated to be 3 (95% CI ratio to be in the range of 0.8122–11.08), indicating the significantly higher infectivity of the p4 virus.

### 3.3. Passive Protection in AG129 Mice

**Figure 3:**
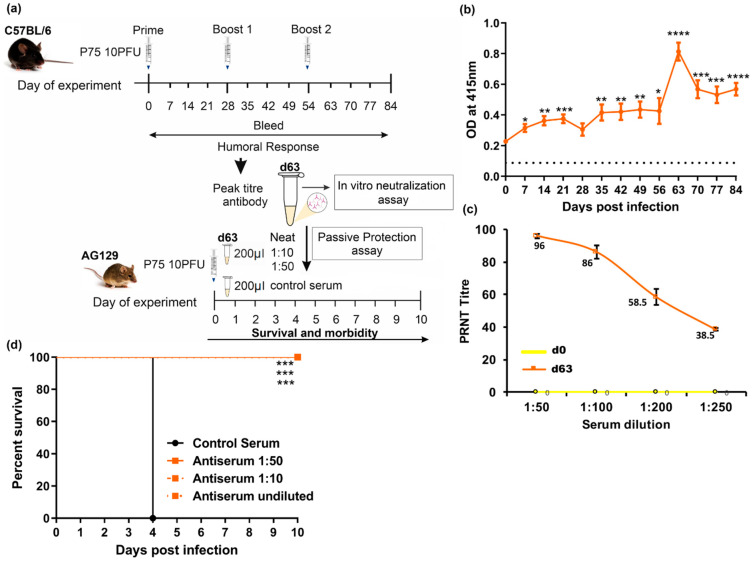
Passive protection assays (**a**) Schematic representation of the experiment design. (**b**) Indirect ELISA for anti-CHIKV antibodies. Two groups of 4- to 6-week-old C57BL/6 mice (n = 6) were immunized thrice with 3-week intervals with 10PFU of CHIKV-attenuated strain virus p75. Serum from individual mice were collected in weekly intervals until 84 days post-infection. Pooled serum at each time point was incubated with recombinant ΔE2 protein coated in 96-well plates, and bound antibody was detected using anti-mouse IgG-HRP. Orange line indicates the absorbance at 415nm on different days post-immunization. Values represent the mean ±SE of duplicate wells. Dotted lines represent the limit of detection, which is half the value of the average OD reading of day 0 samples. * *p* < 0.05, ** *p* < 0.01, *** *p* < 0.001, **** *p* < 0.0001 (dpi vs. d0) (**c**) Plaque Reduction Neutralization Test (PRNT) to evaluate CHIKV-neutralizing antibody. Pooled serum from C57/BL6 mice (n = 6) from day 63 post-vaccination was subjected to PRNT assay and compared with day 0 sera. Heat-inactivated serum was 2-fold serially diluted and incubated with 10^2^ PFU of p75. The sera–virus mixture was added to the Vero cell monolayer for plaque formation. The number of plaques was counted, and a 90% reduction (PRNT_90_) was calculated. The data are presented as geometric mean ± SD. (**d**) Passive protection studies in AG129 mice. Two groups of 3- to 4-week-old AG129 mice (n = 6) were infected subcutaneously with 10PFU of p75 variant followed by immediate intraperitoneal administration of 200 µL of pooled, day-63 post-vaccination serum from immunized animals. Serum was administered either undiluted (orange solid line) or in a 1:10 (orange dotted lines) and 1:50 (orange dashed lines) dilution. Control mice received normal, undiluted mouse serum (black solid line). Mice were monitored daily for survival and morbidity. Survival was compared using Kaplan–Meier survival curves (log rank test). *** *p* < 0.001 (control serum vs. antiserum undiluted/1:10/1:50 dilutions). Antiserum-treated groups (orange lines) are merged, and the percent survival is 100% for 3 dilutions.

A schematic representation of the experiment is depicted in Figure 3a. Initially, we generated the protective antibody containing sera by immunizing 4- to 6-week-old C57BL/6 mice (n = 6) subcutaneously with 10 PFU of the attenuated strain p75. Booster doses were given on day 28 and day 56 of immunization and serum was collected in weekly intervals up to day 84 of immunization. The humoral response was evaluated in an indirect ELISA using a purified ΔE2 CHIKV antigen. We observed that CHIKV-specific antibody levels significantly elevated at day 63, soon after the administration of the second booster dose (Figure 3b). Sera from mice at day 63 post-vaccination (n = 6) had the maximum titer and were pooled. The serum was checked for its ability to neutralize the CHIKV infection in vitro using the plaque reduction neutralization test (PRNT). These antibodies neutralized CHIKV efficiently and a PRNT_90_ was obtained in a 1:75 dilution (Figure 3c) of the serum. 

Further, to evaluate the protection of immune serum in an in vivo infection model, we used adult AG129 mice. AG129 mice are susceptible to both the wild-type and p75-attenuated strain of CHIKV. We used a virus challenge model by infecting two groups of 3–4-week-old AG129 (n = 6) mice with 10 PFU of the p75 virus subcutaneously. It was immediately followed by intraperitoneal administration of antiserum, either undiluted (neat) or in a 1:10 and 1:50 dilution. As shown in Figure 3d, passively transferred antiserum, either undiluted or diluted, conferred full protection in these mice until the end of the observation period of 10 days, by which time all the control animals were dead. Protected mice had no weight loss or any significant clinical signs, while control animals injected with neat normal mouse serum succumbed to infection by day 4 (MDOD = 4 days). The hazard ratio of CHIKV-infected mice with control serum passively administered vs. CHIKV-infected mice plus immune serum passively administered was found to be undefined, as all the animals in the latter group survived for the observation period.

### 3.4. Comparative Whole-Genome Sequence Analysis of the Wild-Type and Attenuated CHIKV Strains to Identify Adaptive Mutations

The wild-type virus p4 as well as p75 were subjected to whole-genome sequencing, to identify the adaptive mutations acquired during passaging of the virus, multiple times in the human cell line. We compared the amino acid changes in the attenuated strain (p75) and wild-type virus (p4) with the first live-attenuated prototype vaccine strain 181/25 and its parent Asian strain AF15561 (Genbank accession number-EF452493). The changes in the amino acids are provided in Table 2. 

One common mutation identified in both attenuated strains (p75 of ECSA genotype and 181/25 of Asian genotype) was the glycine to arginine change in the envelope glycoprotein 2 (E2; G82R). The strain 181/25 differed from its parent virus by having four more substitutions: in nsP1 (T301I), E2 (T12I), TF (C42F) and E1 (A404V) [13]. However, these mutations were absent in the current vaccine strain (p75) under study. There were five novel mutations unique to p75, whereas the corresponding positions in the wild-type p4, 181/25 strain and AF15561 strain had the residues conserved. The positions identified were in nsP1 (R171Q), nsP2 (V740A), nsp3 (N409T), capsid (Q15L) and E2 (T196). The 252 position in envelope glycoprotein E2 of p75 changed from glutamine to histidine (Q252H) as compared to p4, whereas the same position occupied lysine (K) in 181/25 and its parent strain. Another interesting mutation that occurred in the E1 glycoprotein of vaccine strain p75 is mutation of the reversion of alanine to valine (A226V), the critical adaptive mutation of Indian Ocean sub-lineage of the ECSA genotype that contributed to new vector competence in the *Aedes albopictus* species of mosquitoes [90].

## 4. Discussion

There is a definite need for safe and efficient prophylactic vaccines against chikungunya considering the epidemic potential of the virus and the economic burden caused by the disease. Even while the development of new-generation vaccines is being actively pursued (Table 1), classical approaches are still attractive. Among them, vaccines based on live-attenuated strains are preferred for managing chikungunya in developing nations due to their high immunogenicity in smaller doses and long-lasting protection without the need for repeated immunizations. Hence, in the present study, the attempt was to generate a live-attenuated strain of an ECSA-lineage virus, which was predominant in recent outbreaks [91], by serial passaging in cultured cells. We chose the U-87 MG astrocytoma cell line for virus adaptation for multiple reasons. Our earlier study [85] had found that these cells are very susceptible to CHIKV infection as they are interferon-response-deficient due to multiple mutations in the interferon genes [92,93], and they allow virus growth to a high titer. Secondly, we hoped that the adaptation may help us to obtain a virus strain with exclusive infection-specificity to U-87 MG cells, which represent malignant glioblastoma, a deadly cancer with no efficient treatment and poor prognosis, for developing oncolytic virotherapy [94]. While we could obtain a strain satisfying the first objective, we could not achieve the second one as the strain did not have the cell-type specificity as expected. 

Serial passaging in U-87 MG cells resulted in the efficient adaptation of the CHIKV strain. A clear phenotype was evident via the faster vacuolation of cells and cell death in p75 infection as compared to that caused by the un-adapted virus (Figure 1). The presence of the G82R mutation in the envelope protein E2-coding region in the genome, along with the formation of smaller plaques and better replication as compared to the p4 parent strain, points out the efficient mammalian cell adaptation [54]. We identified a total of eight mutations in the structural and nonstructural proteins of p75 in the sequence comparison. Studies using reverse genetics have identified that the attenuation of the CHIK 181/25 strain is by two mutations in the E2 envelope protein: T12I and G82R [15]. CHIKV strains with G82R mutation have altered glycosaminoglycan binding, reduced in vivo replication, the establishment of viremia, and the activation of early inflammatory responses upon infection, thereby regulating virus virulence and the host responses that contribute to the disease outcome [95,96,97]. However, the mutation T12I, which complements attenuation caused by the G82R mutation in strain 181/25, is absent in p75.

We presume that several other mutations, both in the nonstructural and structural proteins, generated during the adaptation, could further contribute to the virus attenuation. These include changes such as nsP1 R171Q, nsP2 V740A, nsP3 N409T, capsid Q15L and E2 T196K. The role of nonstructural proteins in virulence is evidenced by the fact that in a few CHIKV vaccines under development, the portions of key nonstructural protein coding regions are deleted to effect attenuation (Table 1). It was seen that CHIKV ECSA variants isolated from the patients by culturing in mammalian or mosquito cells acquire the nsP1 R171Q mutation at low passage levels; therefore, this should be considered as the fastest occurring adaptive mutation in the genome [98,99,100,101,102,103]. Studies confirm the beneficial effect of nsP1 R171Q in increasing the fitness [104]. Further, the mutations nsP1 R171Q and nsP2 V740A were the key adaptive mutations that were seen in antiviral compound-resistant CHIKV variants [105,106,107]. E2 T196K mutation in the structural protein region seems to be a cell-type-dependent adaption. Its presence increased CHIKV replication in human lung epithelial cell line A549 but imparted a negative effect on virus replication in African green monkey kidney epithelial cells (Vero cells) [108]. Another interesting observation was the reversion of the *Aedes albopictus*-adaptive mutation [90], E1 A226V, in the p75 virus. It indicates the primary requirement of the amino acid alanine in the 226th position of the protein for sustained replication in mammalian cells. Further studies using reverse genetics approaches would be required to reveal the functional relevance of each of these mutations in the ECSA lineage on its role in viral replication and virulence. 

Humoral immune response effectively protects against CHIKV infection, as is evident from previous studies. It was observed that the passive transfer of neutralizing monoclonal antibodies prevents infection or disease symptoms in mice and nonhuman primates [109,110,111,112]. In our study, while all neonatal BALB/c mice pups infected with p4 died, complete survival of p75-infected animals was observed, pointing to its efficient attenuation (Figure 2). However, in AG129 animals, which are absolutely deficient in IFN-mediated antiviral response, infection with p75 was equally as lethal as with p4. This underscored the role of an efficient IFN response in protection against CHIKV [89]. Immune serum generated from p75-vaccinated BALB/c animals (Figure 3b), when administered to AG129 mice, offered complete survival in passive protection studies (Figure 3d). This indicated that the attenuated p75 strain induces an optimal neutralizing antibody response. These results were consistent with earlier findings demonstrating the antibody-mediated protection of AG129 mice against CHIKV 181/25 virus infection [113].

In conclusion, in the present study, we developed an attenuated CHIKV ECSA lineage strain and characterized its efficacy to protect against infection. This strain could serve as a candidate virus for developing newer live-attenuated vaccines against chikungunya. However, in the present set of experiments, we carried out challenge studies with only a homologous virus strain. Further studies with heterologous virus strains, including clinical isolates belonging to multiple lineages, are essential to evaluate the spectrum of protection offered by p75 and exploit the utility of p75 as a strain for vaccine production.

## Figures and Tables

**Table 1:** Current chikungunya vaccine candidates.

Sl. No.	Name of Candidate Vaccine	CHIKV Strain	Vaccine Type	Details of the Candidate	Reference
Clinical Phase 3					
1	PXVX0317/VRC-CHKVLP059-00-VP	37,997; West African	VLP	Structural polyprotein CE3E26KE1 was inserted into pseudotyped lentiviral vectors and transfected into HEK293 cell line forms of VLPs.	[16,17,18,19,20]
2	Δ5nsP3/VLA1553-301	LR2006 OPY1; ECSA	Live-attenuated	Infectious viruses from cDNA clone with the deletion of 60 amino acids in the hypervariable region of the nsP3.	[21,22,23,24]
3	BBV87	IND-06-AP3; ECSA	Inactivated virus	Whole-virus BPL/formalin inactivated vaccine formulated with 0.25 mg aluminum (as aluminum hydroxide).	[25]
Clinical Phase 2					
4	TSI-GSD-218 (181/clone25) Completed	AF15561; Asian	Live-attenuated	Virus strain was attenuated by 11 passages in Vero cells and sequential 18 plaque-to-plaque passages in MRC-5 cells to develop 181/clone 25.	[13,14,15]
5	MV-CHIKV (V184)	ECSA	Virus vectored, VLP	Measles virus vaccine Schwarz 06-46 strain vector expressing VLPs comprising structural polyprotein.	[26,27,28,29,30]
Clinical Phase 1					
6	Formalin inactivated (15561)	AF15561; Asian	Inactivated whole virus	Standard formalin inactivation protocol on virus strain.	[12]
7	ChAdOx1 Chik (CHIK001)	NA	Virus vectored	Replication-deficient simian adenoviral vector expressing the CHIKV structural proteins CE3E26KE1 forms VLPs.	[35,36,37,38]
8	mRNA-1388 (VAL-181388)	NA	mRNA	mRNA encoding CE3E26KE1	[39]
9	mRNA-1944	SL15649; ECSA	mRNA	Lipid nanoparticle-encapsulated mRNA encoding the heavy and light chains of a human CHIKV specific monoclonal-neutralizing antibody, CHKV-24.	[40,41]
Preclinical (Non-Human Primate)					
10	CHIKV-IRES	LR2006 OPY1; ECSA	Live-attenuated IRES	Manipulation of the structural protein expression CHIKV infectious cDNA clone by replacing its subgenomic promoter with IRES from encephalomyocarditis virus.	[42,43,44,45]
11	CHIKV pMCE321	PC-08; ECSA	DNA	Consensus sequences were optimized for Env expression and inserted into pVax1 expression vector and designated as pMCE321.	[46,47,48]
12	EILV-CHIKV	99659; Asian	Chimeric virus	An insect specific alphavirus EILV cDNA clone was designed to a chimeric virus containing the CHIKV structural proteins.	[49]
Preclinical (Mouse model)					
13	RH-CHIKV, RHEV-CHIKV	LR2006 OPY1; ECSA	Live-attenuated	CHIKVs with mutations in non-structural proteins –nsP1 R532H, nsP2 E515V and a double mutant, were investigated for their suitability as-attenuated CHIKV vaccines.	[50]
14	CHIKV-NoLS	LR2006 OPY1; ECSA	Live-attenuated	Mutation in the nucleolar localization sequence (NoLS) in CHIKV capsid protein was characterized for attenuation.	[51]
15	Stop CHIKV, Superstop CHIKV	LR2006 OPY1; ECSA	Live-attenuated	Live-attenuated CHIKV was designed by applying a rational genomic design based on multiple replacements of synonymous codons.	[52]
16	Chikv HR (TM17-2)	37997; West African	Live-attenuated	Host range mutant generated by attenuating cDNA clone of CHIKV via truncating the transmembrane domain of E2.	[53]
17	Heparin sulfate cell culture adapted	LR2006 OPY1; ECSA	Live-attenuated	Virus stock was serially passaged 10 times in triplicate series on CHOK1, pgsA745 or C6/36 cells for deliberate attenuation through envelope glycoprotein mutation.	[54]
18	CHIKV DRDE-06	DRDE-06; ECSA	Inactivated virus	Vero cell culture-derived, formalin-inactivated CHIKV vaccine candidate.	[55]
19	VLP -CHIKV-S27	S27; ECSA	VLP	Structural polyprotein was inserted into a recombinant baculovirus vector and transfected in insect cell line (*Spodoptera frugiperda* cell lines -Sf21 to generate Ac-S27.	[56,57]
20	VLP–CHIKV-37997	37997; West African	VLP	Structural polyprotein was inserted into a recombinant baculovirus vector and is transfected in insect cell line Sf9 to generate AcMNPV-CHIKV37997.	[58]
21	Yeast expressed VLP	DRDE07; ECSA	VLP	Structural polyprotein was inserted into a yeast expression vector and integrated in GS115 strain of *Pichia pastoris* by electroporation.	[59]
22	Δ5nsP3 and Δ6K DNA	LR2006 OPY1; ECSA	DNA	cDNAs of the CHIKV, Δ5nsP3, or Δ6K strain were cloned under the control of the human CMV promoter in DNA-launched Semliki Forest virus replicon (DREP) plasmid which can produce infectious viruses.	[21]
23	CHIKV-NoLS RNA	LR2006 OPY1; ECSA	RNA	In vivo liposome RNA delivery system delivers the self-replicating RNA genome of CHIKV-NoLS directly into mice, allowing the recipient’s body to produce the live-attenuated vaccine particles—de novo production of live-attenuated vaccine in vivo.	[60]
24	p181/25-7 iDNA	TSI-GSD-218; Asian	DNA	iDNA vaccine comprising of plasmid DNA that encode the full-length infectious RNA genome of live-attenuated CHIKV clone 181/25.	[61]
25	iRNA Δ5nsP3; iDNA Δ5nsP3	LR2006 OPY1; ECSA	RNA, DNA	In vitro transfection of iRNA carrying the deletion of 183 nucleotides in the nsP3 (Δ5nsP3) gene generated infectious viruses. iRNA is under SP6 while iDNA is under CMV promotor.	[21,62]
26	CHIKV-sE1 and –SE2	S27; ECSA	Subunit vaccine	C-terminal his-tagged E1 and E2 envelope glycoproteins were produced at high levels in insect cells with baculovirus vectors using their native signal peptides located in CHIKV 6K and E3, respectively.	[56,57,63]
27	rE2p-CHIK	IND-06-AP3; ECSA	Subunit vaccine	E2 gene of CHIKV isolate was cloned in pET15b vector, expressed and purified (rE2p).	[25]
28	rCHIK-E1/E2	DRDE-06; ECSA	Subunit vaccine	The E1 and E2 gene fragment were cloned into a pET28b + vector, expressed and purified.	[64]
29	VEE/CHIKV EEE/CHIKV SIN/CHIKV	LR2006 OPY1; ECSA	Chimeric virus	Chimeric viruses were constructed with VEEV (TC-83 strain) or EEEV (BeAr436087) or Sindbis virus (AR339) as the backbone and the structural protein genes of CHIKV and passaged on Vero cells.	[65]
30	VEE/CHIKV/IRES-C VEE/IRES-CHIKV VEE/IRES-C/CHIKV	LR2006 OPY1; ECSA	Chimeric virus	The above chimeric viruses were modified and made replication dependent on the function of the encephalomyocarditis virus (EMCV) internal ribosome entry site (IRES) and tested three different strategies of IRES-mediated CHIKV structural protein expression.	[65,66]
31	rVSVΔG- CHIKV	S27; ECSA	Chimeric virus	VSVΔG vector expressing CHIKV envelope proteins	[67]
32	CAdVax-CHIKV	LR2006 OPY1; ECSA	Chimeric virus	Inserting structural polyprotein into non-replicating complex Adenovirus vaccine (CAdVax) vectors.	[68]
33	MVA-CHIKV	LR2006 OPY1; ECSA	Chimeric virus	Based on the highly attenuated poxvirus vector modified vaccinia virus Ankara (MVA) expressing the CHIKV CE3E26KE1 structural gene.	[69,70]
34	MVA-6KE1 MVA-E3E2 MVA-6KE1E3E2	S27; ECSA	Chimeric virus	Recombinant MVA vector expressing E3E2, 6KE1, or the entire CHIKV envelope polyprotein cassette E3E26KE1.	[71]
35	E2EP3	NA	Epitope based	KLH-E2EP3 peptide with adjuvant when administered in mice protected against CHIKV.	[72]
36	CHIKV 181/25 CHIKV 181/25- Δ5nsP3	TSI-GSD-218; Asian	Live-attenuated RNA hybrid	Full-length replication-competent attenuated CHIKV genomes are delivered to the site of vaccination using cutting-edge thermostable RNA vaccine delivery technology.	[73]
37	HydroVax-CHIKV	TSI-GSD-218; Asian	Inactivated virus	Site-directed hydrogen peroxide-based inactivation approach which maintains antigenic structures.	[74]
38	TR-S	LR2006 OPY1; ECSA	Trans-amplifying RNA	A trans-replicon (TR) RNA encoding the CHIKV envelope proteins can be amplified by the replicase (which are formed by a non-replicating mRNA encoding for the CHIKV nonstructural proteins) in trans.	[75]
39	E2-E1-LNP	Asian strain	mRNA	mRNA-lipid nanoparticle (mRNA-LNP) vaccine expressing CHIKV E2-E1 antigen.	[76]

**Table 2:** Key amino acid variations in attenuated CHIKV strains.

		Mutations Identified			
Protein	Amino Acid Positions	RGCB 355/KL08-p4 (Virulent)	RGCB 355/KL08-p75 (Attenuated)	AF15561 Asian Lineage Parent Strain of 181/25 (Virulent)	TSI-GSD-218 181/Clone25 (Attenuated)
nsP1	171	R	Q	R	R
	301	T	T	T	I
nsP2	740	V	A	V	V
nsP3	409	N	T	N	N
Capsid	15	Q	L	Q	Q
E2	12	T	T	T	I
	**82**	**G**	**R**	**G**	**R**
	196	T	K	T	T
	252	Q	H	K	K
E1	226	V	A	A	A

(Amino acid position and residues shown in bold are already implicated as virulence-determinants [15]).

## Data Availability

All data from the study have been included in the manuscript itself.
